# Dynamic charging as a complementary approach in modern EV charging infrastructure

**DOI:** 10.1038/s41598-024-55863-3

**Published:** 2024-03-09

**Authors:** Duc Minh Nguyen, Mustafa A. Kishk, Mohamed-Slim Alouini

**Affiliations:** 1 Computer, Electrical and Mathematical Science and Engineering Division, King Abdullah University of Science and Technology (KAUST), Thuwal, 23955-6900 Saudi Arabia; 2 Department of Electronic Engineering, Maynooth University, Maynooth, W23 F2H6 Ireland

**Keywords:** Electrical and electronic engineering, Energy infrastructure

## Abstract

Whether the future of transportation is going to be electric or not is no longer a question. Electric vehicles (EVs) offer several benefits toward global sustainability. However, without a variety of charging infrastructures that cover diverse forthcoming charging needs, the speed of vehicle electrification may be slow and limited. In this study, we investigate the synergistic benefits of traditional charging stations and an emerging alternative, i.e., wireless dynamic charging roads, focusing on their impact on the daily commute of EV users. We center our evaluation on quantifiable metrics, chiefly time and convenience, and deploy computer simulations utilizing authentic transportation datasets from New York City, USA. Our findings underscore that integrating both charging stations and charging roads can substantially alleviate detours for EV users and remarkably reduce additional charging time. Our research provides evidence to encourage researchers, EV manufacturers, urban infrastructure planners, and policymakers to explore future charging infrastructures for EVs, with a notable emphasis on wireless dynamic charging roads.

## Introduction

Sustainable transportation has been one of the world’s most prominent goals for several years, mainly because transportation contributes a significant portion to the global emissions of air pollutants. For example, the United States Environmental Protection Agency (US EPA) reports that the transportation sector is responsible for over 50% of total nitrogen oxides (NO_x_) emissions in the US^1^. In addition, transportation accounts for about 29% of total US greenhouse gas (GHG) emissions, making it the most significant contributor to US GHG emissions^2^. Acknowledging this fact, several governments have expressed a strong commitment to vehicle electrification, i.e., replacing internal combustion engine (ICE) cars with electric vehicles (EVs). For example, the US targets 50% of EV sale share in 2030^3^. The UK plans to totally ban diesel and gasoline cars by the same time^4^.

Thanks to incentives from public officials all over the world, EVs are gaining increasing popularity. In 2022, 10.5 million EVs were sold globally, marking an increase of more than 55% compared to 2021. The global market share of EVs in 2022 is about 13%, with sales that made up 7.2% of total new car sales in the US, 27% in China, and 20.8% in Europe^5^. Even though the rate of EV adoption is encouraging, those small market shares of EVs compared to ICE cars signify that some challenges remain for EVs to be adopted by the general public. Research by Castrol (a British oil company) on nearly 10,000 participants reveals that the tipping points to mainstream EV adoption are a retail price of $36,000 per unit, a charging time of 31 min, and a driving range of 469 km^6^. Currently, on the market, the EV model closest to those tipping points is the Tesla Model 3, which is also the best-selling EV model in the US. It has a starting price of $40,240 and up to 236 km of driving range after 15 min of fast charging. Industry advances like the Model 3 may lead to believe that we are only a few years behind the tipping points, as progress in battery design and charging technology will continue to come. However, the caveat is the availability of those fast-charging stations.

UBS Group (a multinational investment bank and financial services company based in Switzerland) estimates that an American, on average, lives 4 min away from a gas station, but 31 min away from a Tesla supercharging station^7^. To match the 4 min distance of gas stations, Tesla needs to build 30,160 more supercharging stations. A supercharging station typically costs $250,000, so Tesla needs $7.6 billion, roughly 1.5 times its annual profit from 2021^8^. A similar situation is also applied to non-Tesla charging stations, e.g., a typical Electrify America fast charging station costs $350,000 to build^9^. Therefore, the solution to future EV charging infrastructure is not simply adding more fast charging stations. It is essential for policymakers, EV manufacturers, and academic researchers to explore other charging alternatives to accelerate vehicle electrification^10,11^.

A charging solution that has gained considerable attention is dynamic charging, i.e., a technology that enables EV batteries to charge while the vehicle is either parked or in motion. This emerging solution has been explored through theoretical models, prototype demonstrations, and public trials globally^12–14^. The technology addresses key limitations of traditional charging stations, specifically reducing the need for EV drivers to make frequent stops for charging and eliminating the requirement for additional land resources^15^. However, a critical drawback of such systems is the associated cost. While the exact financial comparison varies depending on factors like location and hardware specifications, a kilometer of dynamic charging road is generally significantly more expensive than a traditional charging station^13,16,17^.

**Figure 1:**
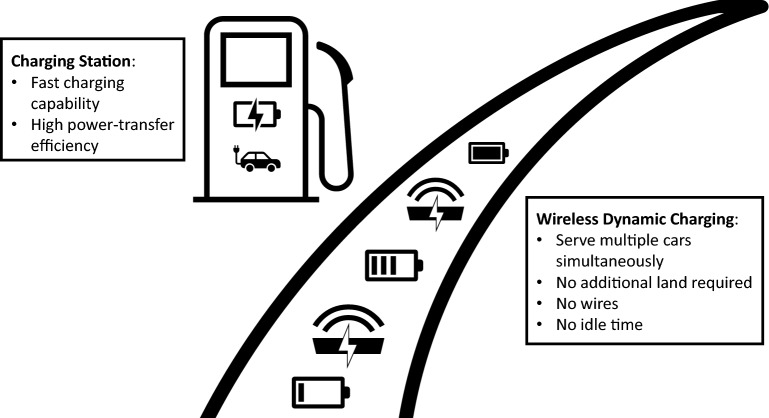
A sketch of future charging infrastructure.

Given the substantial investment and inflexibility of infrastructure changes once implemented, and considering the existing prevalence of charging stations in metropolitan areas, we ask: should future developments focus exclusively on charging stations, dynamic charging roads, or a mix of both? In this paper, we address this question from the perspective of EV drivers, specifically evaluating which option minimizes charging time and enables the most efficient routes. Our contributions can be summarized as follows:

- We examine both existing and emerging charging solutions, i.e., traditional charging stations and dynamic charging roads, and evaluate which approach, or combination thereof, will most benefit EV drivers, as demonstrated in Fig. 1. Our research goes beyond a simple evaluation of the benefits of traditional charging stations and dynamic charging roads. Instead, it addresses the contemporary issue of charging infrastructure shortage, providing a nuanced understanding of how two different charging solutions can coexist and complement each other, which has not been extensively explored in existing literature.
- We employ quantifiable metrics based on factors directly affecting EV drivers: time and convenience. Specifically, we assess the additional time incurred in a trip due to charging requirements and the frequency with which drivers must deviate from their most efficient routes to access charging facilities. This methodology is particularly innovative as it provides a clear, measurable way to assess the benefits and drawbacks of the two different charging infrastructures, thereby aiding in more informed decision-making for future developments.
- We demonstrate our findings using real-world data from New York City, offering empirical insights into the impact of these charging solutions on urban mobility. This empirical approach provides practical insights and adds a layer of authenticity and applicability to our findings, which are valuable for urban planners, policymakers, and EV manufacturers.

To the best of our knowledge, our work is one of few attempts that investigate the impact of both types of charging infrastructure on urban mobility, from the EV drivers’ perspective. The implication and significance of our research can be further detailed below.

- Insights into Urban Planning and Policy Making: The findings of our study are particularly relevant for policymakers and urban planners. They provide empirical insights into how different charging infrastructures can benefit EV drivers in their urban commute. This aspect of our research contributes to a more informed decision-making process for sustainable urban development.
- Advancing the EV Infrastructure Development: Our work contributes to the ongoing EV infrastructure development by highlighting the trade-offs and synergies between two different types of charging technologies. This is crucial for advancing the field and for the practical implementation of these technologies in various urban settings.
- Guiding Future Research and Technological Innovation: The implications of our findings extend to guiding future research and technological innovation in EV charging solutions. By identifying the strengths and limitations of current technologies, our research paves the way for further innovations in this area.
- Global Relevance and Applicability: Given the global push towards electric vehicles and sustainable transportation, our study’s findings have wide-reaching relevance and applicability. They provide insights that are valuable not just for EV drivers, but also for a broader audience including environmental researchers, transport engineers, and government bodies involved in transportation and environmental policy.

The table of notations used in our work is shown in Table 1.

**Table 1:** Summary of notations.

Notation	Description
*r*	Distance (in meters) from the city center
$r_{\textrm{min}}$	Lowest value of *r* at which the power law is obeyed
$\alpha$	Positive constant that describes the rate at which the population and traffic densities decrease

## Related work

In the literature, several studies exploring the implications of deploying dynamic charging systems for EVs have been introduced. One key observation is that dynamic charging roads significantly extend the driving range of EVs in urban settings when assessed using standard urban driving cycles like the US Federal Test Procedure 72 (FTP 72) or the European Union Directives ECE-EUDC^18^. These systems also elevate the battery charge levels when installed along highways, as evaluated by the US HighWay Fuel Economic Test (HWFET) and Mountain-Energy Expensive Driving Cycle (MEEDC), despite requiring more extensive infrastructure^18^.

Additionally, a study using real-world mobility data from Lisbon, Portugal, revealed that at least 15% more drivers could transition to EVs if dynamic charging were accessible, as compared to relying solely on stationary charging systems^19^. Experiments by Lukic et al.^20^, featuring different vehicle types equipped with small battery packs, underscored the drastic enhancement in driving range achievable with minimal dynamic charging infrastructure in urban areas.

Furthermore, an analysis focusing on Sweden indicated that the most cost-effective deployment of conductive charging roads accommodated both heavy-duty and small private vehicles^21^. Other research proposed a framework utilizing wireless chargers to minimize taxi idle times and facilitate seamless operation^22^. Environmental impact assessments of dynamic charging roads have also been conducted, evaluating lifecycle environmental costs^23^.

Studies have also explored network considerations, such as the 24-h impact of fast static and dynamic inductive chargers on power distribution systems in Greece^24^, and the load profile of dynamic wireless charging on a modified IEEE 13-bus network^25^. Regulatory concerns and health and safety guidelines in the context of wireless charging have been examined^26^, along with evaluations of the magnetic field emissions from wireless power transfer systems^27^.

Lastly, a cost analysis comparing stationary, quasi-dynamic, and dynamic wireless charging systems found that dynamic charging is financially favorable when EV battery costs are high but infrastructure costs are low, whereas static wireless charging is more advantageous under the opposite cost structure^28^.

Unlike previous studies, which often evaluate dynamic charging systems in isolation or focus solely on technological aspects, our method considers a comprehensive urban mobility perspective. This includes assessing the collective impact of both traditional and dynamic charging solutions on EV drivers within an existing urban infrastructure.

Moreover, our method evaluates not only the individual merits and limitations of each charging infrastructure but also their combined synergistic potential. This is a significant departure from existing studies that typically assess these systems separately, without considering their interplay within a comprehensive urban mobility framework.

In addition, the evaluative criteria employed in our study are multifaceted, incorporating both quantitative and qualitative aspects of EV usage, such as convenience and route optimization. This nuanced approach sets our research apart from conventional studies, which often concentrate solely on cost-effectiveness or technological capabilities.

Furthermore, our analysis is grounded in real-world data from New York City, focusing on practical metrics such as charging time and route efficiency. This user-centric perspective enriches the existing literature by shifting the focus away from purely economic or technological considerations. It thus serves as an invaluable resource for urban planners and policymakers when making data-driven decisions about future investments in EV infrastructure.

Finally, the valuation method is designed to yield insights that are directly applicable to policymakers and automotive manufacturers, bridging the gap between academic research and practical implementation. This user-centric approach is less common in traditional methodologies, which may not directly address the practical considerations needed for policy-making and urban planning. Accordingly, our work emerges as a well-rounded and practical guide, aiming to make a significant impact on both scholarly research and the practical implementation of sustainable urban mobility solutions.

## The two complementary solutions to EV charging

### Charging stations

Charging stations are currently the most popular means of charging EVs and are likely to stay prominent in the future because of their advantages. They have a high power-transfer efficiency, low maintenance requirements, and the ability to fast charge. However, charging stations possess multiple shortcomings that motivate the development of new charging alternatives. First, charging stations add great pressure to the power grid, especially during peak times such as the rush hours^29^. Second, in metropolitan cities, spaces for charging stations are extremely limited. Setting aside land for charging stations is unlikely to be profitable, given the lower utilization rate. Each EV is likely to stop for half an hour or more to charge. Thus, a charging station can serve much fewer cars in a day compared to a gasoline station. Even though efforts have been made to install charging stations in existing parking lots, another problem is that EV users must pay for parking on top of the charging price. In addition, the EV market lacks a global standard for charging ports, which tend to vary across geographies and models. For example, for DC fast chargers in North America, the three connector types currently in use are CHAdeMO, CCS, and Tesla proprietary connector^30^. This means EV owners have even more limited choices when choosing a place to charge their EVs.

### Dynamic charging

Dynamic charging refers to an energy-transfer system capable of charging EV batteries while at rest or in motion. It can be divided into two categories: conductive solutions and inductive solutions. With conductive dynamic charging, EVs can charge their batteries with conductive contacts, e.g., attaching to overhead/on-road/side wires using a pickup arm, similar to rail and trolley-bus systems. While with inductive dynamic charging, electric power can be transferred to EVs electro-magnetically through an air gap up to a few meters, completely eliminating the need for wires. Both conductive and inductive charging solutions have been thoroughly studied in several research institutes around the world^12^. In the industry for conductive dynamic charging, companies such as Scania, a major Swedish manufacturer of heavy vehicles, is conducting a pilot for long haul electrified trucks to be charged by overhead wires on electric roads^31^. The trucks are able to attach to and detach from the wires on demand to overtake vehicles traveling in the same direction, releasing zero emissions from start to finish. In Sweden, a startup named Elonroad has installed the first kilometer of conductive charging road in the city of Lund^32^. In this project, the testing EVs are modified to use sliding contacts to touch the power plates installed on the road for power pickup. However, conductive charging suffers from a few significant drawbacks compared to wireless solutions, e.g., low alignment flexibility, difficulty of use, and limited automation^12^. Therefore, in this paper, we focus on wireless dynamic charging solutions.

Wireless dynamic charging is a system of charging pads installed under the roads and receivers at the bottom of EVs that allow the complete wireless power transfer on the go. The system is designed to overcome the limitations of charging stations and conductive charging solutions. For example, it completely removes the wires, distributes the load on the power grid, serves multiple cars simultaneously, requires no additional land resources, and reduces the idle time stopping to recharge^15^. Moreover, the dynamic charging system does not carry expensive operational costs, as it can be controlled to automatically turn on only when EVs with valid credentials are detected. Therefore, dynamic charging has great potential to support vehicle electrification to reduce GHG emissions and improve air quality.

Both light and heavy-duty commercial vehicles, e.g., taxis, buses, and trucks, will be the first class of vehicles that benefit from the dynamic charging systems. Since such vehicles need to maximize their time in service, charging wirelessly while in motion will directly convert to more service hours, bringing more profit. In addition, research shows that frequent short charging cycles, as in the case of using dynamic charging, can increase battery life by nearly three times^33^. Until now, batteries are still the single most expensive components in EVs^34^. Therefore, the reduction in battery size and prolonged battery life will cut the maintenance cost of those vehicles. In the long run, the cost of using dynamic charging may even be lower than charging stations, especially if a company invests in both its commercial electric fleets and charging infrastructures^17^. From an environmental point of view, smaller batteries on EVs also reduce the amount of battery waste when those batteries reach their end of life.

Currently, the main concern of such system is cost. The dynamic charging system requires infrastructure investment, i.e., charging pads, road construction, as well as vehicle investment, i.e., receivers in EVs. However, there are several funding opportunities for the technology, e.g., grants for EV transformation from local governments. In addition, to enhance scalability, the private sector can also partner with the relevant public agencies to commercialize the dynamic charging system, offering charging as a paid service to EV drivers. To this end, researchers and engineers have proposed several authentication and payment models for the wireless dynamic charging system^35–37^.

Given the valuable opportunity of dynamic charging to support the electrification of various vehicle types, e.g., personal cars, buses, school and airport shuttles, trucks, and seaport vehicles, it is essential that the feasibility and benefits of such system are well understood and embraced. In the next section, we elaborate on the background and current development of the dynamic charging system.

## Dynamic charging system summary

### Proof of concept

**Figure 2:**
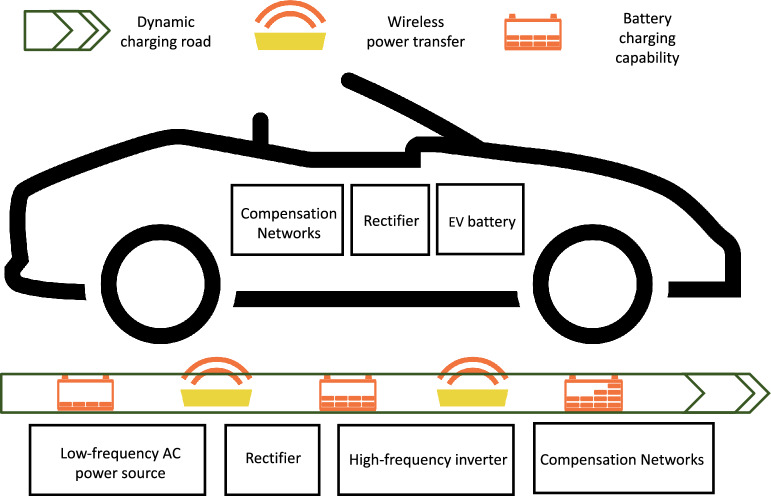
Key components of a wireless dynamic charging system.

The technology behind dynamic charging is wireless inductive power transfer, which allows power delivery through an air gap of two magnetically-coupled coils: one installed on/under the road and the other at the bottom of an EV^13^. The transmitter on/under the streets often consists of a rectifier, a high-frequency inverter, a primary compensation network, and a primary coil, as shown in Fig. 2. The rectifier first converts the low-frequency AC power from the power grid into DC power. Then, the DC power is converted to high-frequency AC power by the inverter to pass to the primary compensation network. The compensation network is a system of inductors, capacitors, and resistors. It ensures sufficient wireless power delivery between the two magnetically-coupled coils, which are susceptible to high leakage due to their long distance. The receiver at the bottom of an EV features similar components: a secondary coil, a secondary compensation network, a rectifier, and a battery system. On the receiver side, after the received power is maximized through the secondary compensation network, it is rectified into DC power to charge the EV battery. Different components of an inductive power transfer have been extensively studied in the literature. For a detailed review of the designs of each element, we refer the readers to a comprehensive survey in^38^.

### Prototypes

Several research institutes have demonstrated prototypes of wireless stationary and dynamic charging systems^13^. For example, in KAIST, South Korea, six generations of dynamic charging systems have been deployed and tested since 2009^39^. The first generation system powered a golf cart with an output power of 3 kW at a 1-cm air gap. Fast forward to the fourth generation, the system was able to deliver 60kW power at a 20-cm gap to electric buses. Next, in the fifth and sixth generations, the overall cost of the system was further reduced, and the seamless integration of static and dynamic charging systems was studied, respectively. After that, those electric buses were deployed on KAIST campus and the cities of Gumi and Sejong in South Korea. In the US, researchers at the Oak Ridge National Laboratory have focused on some aspects of the wireless power transfer systems, such as coil design, inverter regulation, and vehicle integration. In 2013 and 2018, the team demonstrated wireless stationary charging on popular consumer vehicles such as the Toyota Prius^40^ and Toyota Rav4^41^, respectively. The system for the Toyota Rav4 achieved a power of 20kW at a 162-mm air gap with a DC-to-DC efficiency of 95%.

### Market deployment & demonstration

The current EV market has seen both wireless static and dynamic charging in production from industry leaders worldwide. In the US, the company Electreon will deploy its dynamic charging system, i.e., charging roads, at several test sites across the states, e.g., Utah and Michigan, in 2022 and 2023, respectively^42,43^. In Europe, Renault Group is leading an extensive demonstration of dynamic charging in cities such as Amsterdam, Paris, Turin, Tallinn, and Zaragoza^44^. The project coordinates 33 partners and has a budget of $20 million. In Nottingham (United Kingdom), five wireless ground charging spots have been deployed at the main taxi rank near a train station^45^. The EV taxis can simultaneously top up the batteries while waiting for passengers. As the taxis move forward in the rank, charging is automatically resumed on the next pad. At the end of a day, taxi drivers will receive the charging bills through an app on their phones. This project is named the Wireless Charging of Electric Taxis (WiCET), which is supported by several organizations such as Innovate UK, Coventry University, Nottingham City Council, Shell Research Limited, and Lumen Freedom. With the current market development, dynamic charging has a high potential to become a critical framework in the future charging infrastructure for EVs.

## Case study

### Assessment metrics

In this section, we use computer simulation to illustrate the impact of wireless dynamic charging roads on urban mobility. Our goal is to determine if a future with (1) only charging stations, or (2) a combination of charging stations and dynamic charging roads, will be more beneficial to EV drivers. Specifically, we focus on how those charging facilities can ease the range anxiety problem, enabling EV drivers to finish their trips with sufficient energy. Next, we aim to see which scenario adds the least extra charging time to drivers’ trips. To this end, we simulate the following two metrics:

#### Metric 1

(Percentage of trips that detoured) It is the number of trips that have to detour from the shortest routes to find a charging facility (due to insufficient energy) divided by the total number of trips.

#### Metric 2

(Percentage of extra time added to a trip) It is the percentage of the extra time added to a trip due to charging at a charging station. For example, if a trip takes 50 min and charging takes 10 min, then the percentage of added extra time is $\frac{10}{50} * 100\% = 20\%$. Since wireless dynamic charging roads can power EVs while driving, no extra time is added to trips due to charging.

The first metric measures the impact of the charging infrastructure on the overall efficiency of travel routes. It helps to determine how well the charging network is integrated into the city and if it aligns with the natural flow of traffic. The second metric is critical to drivers of commercial electric vehicles such as delivery vans or taxis, as this metric directly relates to operational costs and customer experience. For example, for a taxi driver, a reduced added time implies that he or she can fit more trips in a day, generating more profit. The same metric for a delivery van driver means more orders can be fulfilled.

### Datasets

To reflect the actual traffic, we utilize a six-month trip record dataset of yellow taxis from July 2019 to December 2019 in New York City (NYC). Yellow taxis are a signature of NYC and are the only serviced vehicles allowed to take street hails from every city borough. The dataset contains over 33 million records and is made available online by the New York City Taxi and Limousine Commission (TLC)^46^. In the dataset, NYC is divided into 263 zones corresponding to the NYC Department of City Planning’s Neighborhood Tabulation Areas. Then, the starting and ending points of each trip are documented based on those zones. Since the traveled routes are not provided to maintain privacy, we assume the shortest driving routes for all those trips. We choose NYC since the city has a rich and reliable dataset on historical trip records. However, our simulation and analysis can be easily extended to other metropolitan cities where relevant datasets are available. For the road networks of NYC, we rely on data supplied by OpenStreetMap^47^.

### Methodology

To compare two scenarios of (1) only charging stations, and (2) a combination of charging stations and dynamic charging roads, we assume a baseline of fifty level-two charging stations, since those are much cheaper than DC fast chargers and are likely to scale faster than DC fast chargers in the near future. In addition, a baseline scenario with existing infrastructure provides a useful point of comparison to measure the impact of any additional investments in charging stations or dynamic charging roads. It is also representative of many current urban settings where initial investments in EV charging infrastructure have already been made. Then, we investigate two future scenarios. One is when the city is equipped with another fifty charging stations. The other is when the city adds certain kilometers of dynamic charging, as summarized in Table 2.

**Table 2:** Summary of compared scenarios.

Scenarios	Description
Baseline	50 level-2 charging stations
Future scenario 1	Adding another 50 charging stations
Future scenario 2	Equipping 0.02%, 0.1%, 0.3%, 1%, 3%, and 5% of the roads in NYC with dynamic charging systems, Corresponding to 25, 57, 118, 385, 822, and 1226 kilometers of dynamic charging roads, respectively

Charging stations and dynamic charging roads are typically distributed in cities based on the spatial patterns of population density and socio-economic activities. Previous research has shown that in modern metropolitan areas, both population and traffic densities are highest at the city center and decline with increasing distance from the center. This decline often follows a power-law function, denoted as $g: {\mathbb {R}} \mapsto [0,1]$, expressed as:

1 $$\begin{aligned} g(r) = {\left\{ \begin{array}{ll} (\frac{\mid r \mid }{r_{\textrm{min}}})^{-\alpha } &{} \mid r \mid > r_{\textrm{min}}\\ 1 &{} \mid r \mid \le r_{\textrm{min}}, \end{array}\right. } \end{aligned}$$

where $r_{\textrm{min}}>0$ is the minimum value of *r* at which the power law holds, and $\alpha$ is a positive constant that describes the rate at which the population and traffic densities decrease^48,49^.

In our simulations, we use the same power-law function to determine the location of charging stations and dynamic charging roads under different scenarios. Specifically, we employ Eq. (1) to calculate the likelihood that a particular street intersection will host a charging station or that a specific road segment will be equipped for dynamic charging. The proximity of a road or intersection to the city center influences this likelihood, with closer locations having a higher probability of receiving such infrastructure.

We set $r_{\textrm{min}}$ at 200 m for the simulations. The value of $\alpha$ is chosen such that it aligns with the expected densities of charging stations or lengths of charging roads as described in the scenarios in Table 2. For instance, in the baseline scenario, we select $\alpha$ so that the total expected number of charging stations, summed across all intersections, matches the target value of 50. In a future scenario where we aim to install certain kilometers of dynamic charging roads in New York City, $\alpha$ is determined through multiple simulations to ensure that the average total length of the dynamically charged roads approximates the proposed kilometers.

We assume that all taxis in the experiment are EVs and that all trips start with a battery level uniformly distributed between 10% and 20%. This range mimics a real-world scenario where taxis may not always start a shift fully charged but are not so low on charge that they cannot complete short trips. It also introduces a practical level of stress on the charging infrastructure and lets us focus on the impact of charging infrastructure on trips that require charging.

The charging behavior of EV drivers, e.g., choosing when, for how long, and at which station to charge, in practice, is complicated and may depend on several factors such as personal schedules, charging prices, and station availability and accessibility^50^. In this case study, we assume a general behavior that an EV driver will stop to charge for 20 min if there is a charging station on their routes and the EV battery level is under 10%. This assumption allows all drivers to act uniformly, making it easier to model and interpret the behavior across a fleet of taxis. Another option could be to assume that a driver will charge until the battery reaches a sufficient level for the trip. However, since we are particularly interested in the extra added time to a trip due to charging, i.e., Metric 2, a fixed 20-min charge would lead to more frequent stops as the number of stations increases, providing a clearer mechanism to study the impact of the increased number of charging stations on this metric. In the model where the driver charges “until sufficient,” adding more charging stations might not substantially change the “extra added time” because drivers would simply fill up as needed, regardless of the number of stations. A fixed 20-min charge also better reflects the operational constraints of a taxi service, where time off the road directly translates to lost revenue.

Since our experiment aims to find the aggregated impact over millions of trips, we assume an average driving speed inside NYC of 20 km/h and a constant energy consumption model, i.e., the amount of energy that an EV consumes equals a fixed rate times the traveled distance. To be specific, in the simulation, we assume an energy consumption rate of 0.186 kWh/km, which is close to the EPA-estimated energy consumption rate of the Nissan Leaf and the Chevrolet Bolt (two of the best-selling EVs models in the US). In addition, for charging facilities, we assume an output power of 6 kW for all level two charging stations and 20 kW for dynamic charging roads based on surveys of popular charging stations and dynamic charging systems^38,51^. We also assume a constant charging model, i.e., the amount of energy charged equals the time that the EV is plugged in or is traveling on dynamic charging roads times the output power of the charging facility. In reality, the energy consumption and charging rates will depend on several determinants such as weather conditions and driving behaviors. However, since the simulation is performed on a large number of trips spanning an extended period of time, we adopt a simple assumption of constant energy consumption and charging models.

**Figure 3:**
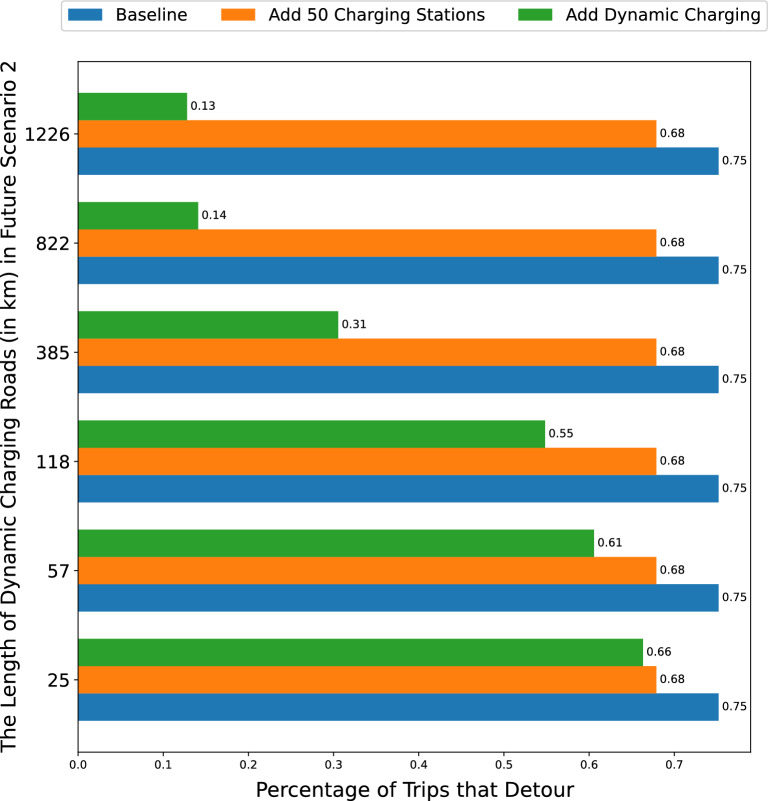
The percentage of trips that detour in three scenarios with various kilometers of dynamic charging roads.

Using the road network dataset from OpenStreetMap, we simulate each scenario from Table 2. For the baseline and scenario 1, 50 and 100 charging stations are generated based on the probability in Eq. (1), respectively. For scenario 2, we investigate the cases when 5%, 3%, 1%, 0.3%, 0.1%, and 0.02% of the roads in NYC are equipped with dynamic charging systems, corresponding to 1226, 822, 385, 118, 57, and 25 km of charging roads, respectively.

### Empirical results

We analyze yellow taxi trip data to assess two key performance metrics under varying scenarios. The first metric, i.e., Metric 1, measures the percentage of trips that must detour to recharge before reaching their destination. These detours occur because the vehicle exhausts its battery before completing the shortest path. Figure 3 shows the percentage of trips that detour according to the varying numbers of kilometers of dynamic charging roads. We observe that implementing 25 km of dynamic charging roads has a slightly greater impact in reducing detours than installing 50 level-2 charging stations. Specifically, both scenarios cut the detour rate by approximately 0.1% compared to the baseline scenario. Calculating the relative differences, the addition of 50 charging stations reduces the detour rate by $\frac{0.75-0.68}{0.75} * 100\% = 9.3\%$, while 25 km of dynamic roads result in a $\frac{0.75-0.66}{0.75} * 100\% = 12\%$ reduction.

While the costs of constructing charging stations and charging roads will vary by city due to factors such as land and construction costs, our results serve as a general guide to their potential benefits. Thus, specific cost considerations are left to local urban planners and policymakers in individual cities.

**Table 3:** Effect of dynamic charging road expansion on Detour reduction.

Kilometers of dynamic charging roads	Charging road increase factor	Metric 1 Reduction	Metric 1 decrease factor
25 (baseline)	1	12%	1
57	2.3	18.7%	1.6
118	4.7	26.7%	2.2
385	15.4	58.7%	4.9
822	32.9	81.3%	6.8
1226	49	82.6%	6.9

Table 3 presents how increasing the length of dynamic charging roads correlates with reductions in Metric 1. Importantly, this relationship is positive but non-linear with a diminishing return, indicating that while the initial expansion of dynamic charging infrastructure significantly mitigates Metric 1, the effectiveness of further expansion lessens with scale. For instance, early enhancements, such as increasing from 25 to 57 km, significantly improve the reduction factor from 1 to 1.6. However, extensive expansions, from 822 to 1226 km, yield a minimal increase in the reduction factor from 6.8 to 6.9, despite a substantial investment in infrastructure. This pattern suggests that, while costs remain high, it may be more cost-effective to install dynamic charging systems on a limited number of high-traffic routes. However, as the technology matures and costs decline, broader implementation may become viable. Urban planners and policymakers should weigh these considerations carefully when deliberating on the expansion of charging infrastructure.

**Figure 4:**
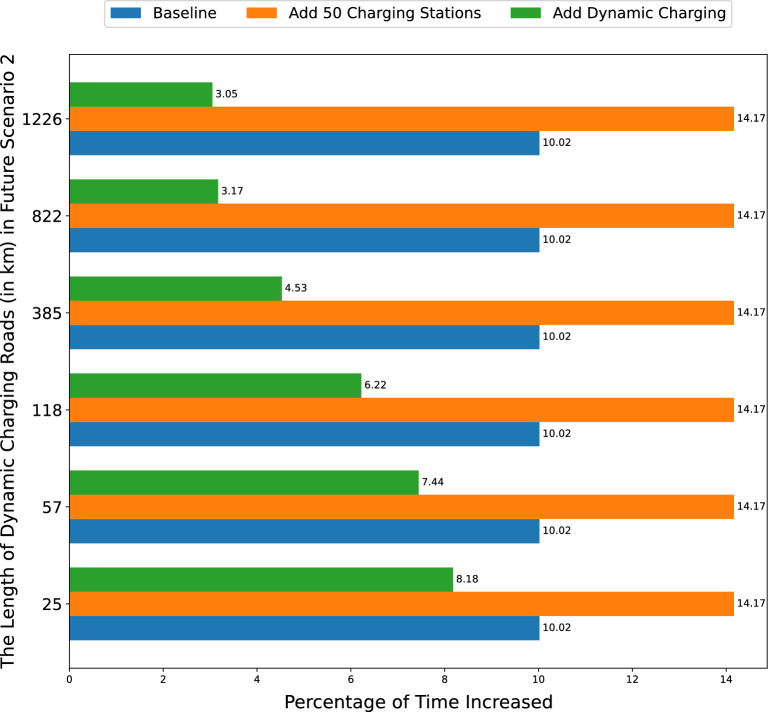
The percentage of time increased in a trip in three scenarios with various kilometers of dynamic charging roads.

In Fig. 4, we present the percentage of extra time added to a trip due to charging, i.e., Metric 2. One can see that when another 50 charging stations are introduced, i.e., future scenario 1, the trips see a significant 41% surge in added charging time. In contrast, the integration of dynamic charging roads leads to a decrease in this added charging time. This discrepancy can be understood in conjunction with the findings from Fig. 3, which demonstrates that both added charging stations and charging roads reduce the necessity for detours. Specifically, with the increased availability of charging stations, the detour rate diminishes as EV drivers have more access points to recharge. Consequently, they spend longer periods charging, ensuring their vehicles have enough energy to complete their journeys. This situation presents an intriguing trade-off, i.e., the advantage of fewer detours is offset by the extended charging durations.

Conversely, dynamic charging roads offer a dual advantage, i.e., they reduce both the detour rate and the charging duration. When 25, 57, 118, 385, 822, and 1226 km of NYC roads are embedded with dynamic charging systems, the extra charging time drops by 18.4%, 25.7%, 37.9%, 54.8%, 68.4%, and 69.6%, respectively, compared to the baseline. However, the introduction of charging stations leads to a 41.4% increase in this metric. It implies that dynamic charging roads have a clear advantage over traditional charging stations in efficiency and user convenience.

The insights from our study are invaluable for urban planners and policymakers. As cities consider resource allocation for EV infrastructure, our findings suggest that, in the long run, dynamic charging roads might offer more tangible benefits compared to conventional charging stations. Moreover, our results could be leveraged to drive EV adoption rates. Assurances of minimized detours and reduced charging times could make EVs an even more appealing proposition for the modern commuters.

## Future research directions & summary

### Economic analysis of dynamic charging roads

While our study underscores the advantages of dynamic charging roads, an economic analysis could further illuminate their cost-effectiveness. If dynamic charging roads can deliver superior benefits at comparable or even diminished costs, they might represent an economically sensible choice. For urban centers that are not yet prepared for a transition to dynamic charging roads, the insights from this study can inform the strategic placement of charging stations, focusing on high-traffic areas, thereby mitigating the extended charging times. Lastly, the evident benefits associated with dynamic charging roads could motivate further research, drive innovation to enhance efficiency, reduce installation costs, and inspire novel methodologies for their rapid deployment.

### Vehicle-to-grid (V2G) support

A potential development of wireless dynamic charging is building its support for vehicle-to-grid (V2G). As we prepare for the mass adoption of EVs, concerns about the stability of the power grid have been frequently raised. To address this problem, V2G technology capitalizes on the large capacity of EV batteries as a source of energy storage to support the grid during times of high demand. In addition, as we transit into sustainable energy sources such as wind and solar, V2G technology also enables EVs to transfer power to the grid during periods of low wind and little sunlight. Some cities and companies have embraced this concept of bi-directional charging. For example, the city of Utrecht (Netherlands) now has hundreds of bi-directional EV chargers^52^. Hyundai, Nissan, and Volkswagen have started to produce models with bi-directional charging capability. Wireless dynamic charging can make V2G technology more available and more convenient. In addition, although enabling bi-directional charging on wired solutions requires expensive additional hardware, wireless charging only needs minor changes to make this technology available.

### Scaling wireless dynamic charging solutions

While significant progress on the proof-of-concept, prototypes, pilot programs, and early commercialization of wireless (dynamic) charging has been introduced, there is still plenty of room for improvement so that the technology can scale. First, researchers and engineers need to optimize the critical components of the dynamic charging system, such as charging pads and power coils, so that the total system cost is competitive compared to that of charging stations. Second, there needs to be a consensus among EV manufacturers and dynamic charging facility providers to ensure the same charging standard across different brands. Third, practical deployment issues such as safety, grid integration, scheduling the power of dynamic charging power rails, and the location of charging roads should be studied by infrastructure & construction companies and urban policy makers. Fourth, the local government of metropolitan cities needs to come up with regulations that facilitate the construction and operation of dynamic charging.

### Integration with smart city infrastructure

Future research could explore the integration of dynamic charging solutions with broader smart city infrastructure. This involves understanding how dynamic charging can be synchronized with other smart technologies, like traffic management systems and renewable energy sources, to optimize energy consumption and reduce congestion.

### Environmental impact assessment

Another critical area for future work is a detailed environmental impact assessment of dynamic charging solutions. This includes life cycle analyses to quantify the environmental benefits and potential drawbacks compared to traditional charging solutions.

### User experience and social acceptance studies

Research into user experience and social acceptance of dynamic charging roads is essential. Understanding public perception, potential barriers to adoption, and strategies to increase acceptance could significantly influence the success of these technologies.

### Conclusion

In this study, we critically examined the optimal focus for future developments in electric vehicle (EV) charging infrastructure: should the emphasis be on conventional charging stations, dynamic charging roads, or a strategic blend of both? Drawing upon datasets from New York City’s street networks, our findings illustrated that, although an increased number of charging stations provide EV drivers with enhanced charging accessibility, this comes with the unintended consequence of prolonged wait times. In contrast, dynamic charging roads present a distinctive advantage. They not only markedly diminish the need for detours but also significantly reduce the supplementary charging duration. This highlights their potential superiority over traditional charging stations in terms of operational efficiency and end-user satisfaction. Consequently, we advocate for a collaborative approach involving academic researchers, EV manufacturers, urban infrastructure specialists, and policymakers to collectively steer the development and deployment of innovative charging solutions, with a notable emphasis on wireless dynamic charging roads.

## Data Availability

The trip record dataset of yellow taxis in NYC analysed in the current study is publicly available online from the website of the New York City Taxi and Limousine Commission (TLC)^46^. The road network dataset of NYC is publicly available online from OpenStreetMap^47^.
